# Central nervous system germ cell tumor, an archetypal AYA tumor and a model for pediatric and neuro-oncology collaboration, review from the EURACAN domain 10 group

**DOI:** 10.3389/fonc.2022.971697

**Published:** 2022-09-29

**Authors:** Cecile Faure Conter, Gabriele Calaminus, James Nicholson, Ahmed Idbaih, Khê Hoang Xuan, Alexandre Vasiljevic, Giovanni Morana, Alexandru Szathmari, Thankamma Ajithkumar, Didier Frappaz

**Affiliations:** ^1^ Institute of Pediatric Hematology and Oncology, Lyon, France; ^2^ University of Bonn, Bonn, Germany; ^3^ Department of Paediatric Oncology, Cambridge University Hospitals NHS Foundation Trust, Cambridge, United Kingdom; ^4^ Sorbonne Université, Institut du Cerveau - Paris Brain Institute - ICM, Inserm, CNRS, AP-HP, Hôpital Universitaire La Pitié Salpêtrière, DMU Neurosciences, Paris, France; ^5^ Centre de Pathologie et Neuropathologie Est, Hospices Civils de Lyon, Lyon, France; ^6^ Department of Neurosciences, Neuroradiology Unit, University of Turin, Turin, Italy; ^7^ Department of Neurosurgery, Hôpital Femme Mère Enfant, Hospices Civils de Lyon, Lyon, France; ^8^ Department of Clinical Oncology, Cambridge University Hospitals NHS Foundation Trust, Cambridge, United Kingdom

**Keywords:** adolescents and young adults (AYA), trial recruitment, collaboration, central nervous system germ cell tumours, euracan

## Abstract

**Simple Summary:** Adolescents and young adults (AYA) with cancer often fall through gaps between children’s and adults’ cancer services. They are consequently under-represented in clinical trials, and their survival is often inferior to that of children or adults with the same tumor type; in this paper, we use the example of central nervous system germ cell tumors (CNS-GCT), as a model of AYA tumor to illustrate this challenge. We describe how we have built bridges between pediatric and adult oncology, how this can apply to other types of brain tumors, and discuss ways to promote cancer care in the AYA population.

Adolescents and young adults (AYA) with cancer are under-represented in clinical trials and have thus not benefited from the same improvement in outcomes as either younger or older patients. Central nervous system germ cell tumors (CNS-GCT) represent an ideal model of AYA tumor as their incidence peaks during adolescence and young adulthood. Since the early 90’s, SIOP (International Society of Pediatric Oncology) has launched two successive European trials: SIOP CNS-GCT96 (January 1996 to December 2005) and SIOP CNS-GCTII protocols (October 2011 to July 2018), for CNS-GCTs. With the removal of the upper age limit in the SIOP CNS-GCTII trial, and closer collaboration between pediatric and adult oncologists within AYA multidisciplinary tumor boards, the proportion of adults enrolled in France has dramatically increased over time. The current article will use the example of CNS-GCT to illustrate how to build a bridge between pediatric and adult oncology, how this can apply to other types of brain tumors, and how to promote cancer care in the AYA population.

## Introduction

The early evolution of pediatric oncology care as a separate specialty happened in relative isolation from adult practice. But it is now clear that adult and pediatric oncology have a great deal to learn from each other and are increasingly combining efforts for those diseases affecting both populations. Collaboration is critical to addressing common challenges. Adolescents and Young Adults (AYA) with cancer often fall through gaps between pediatric and adult cancer services. They are consequently under-represented in clinical trials, and their survival remains poorer than that of children or adults with the same tumor type (1). Increased participation in clinical trials of AYA patients is, therefore, of crucial importance. This article will use the example of Central Nervous System Germ Cell Tumor (CNS-GCT), as a model of an AYA tumor. It will illustrate how to build a bridge between pediatric and adult oncology, how this can apply to other types of brain tumors, and discuss ways to promote cancer care in the AYA population.

## Central nervous system germ cell tumor

**Figure 1:**
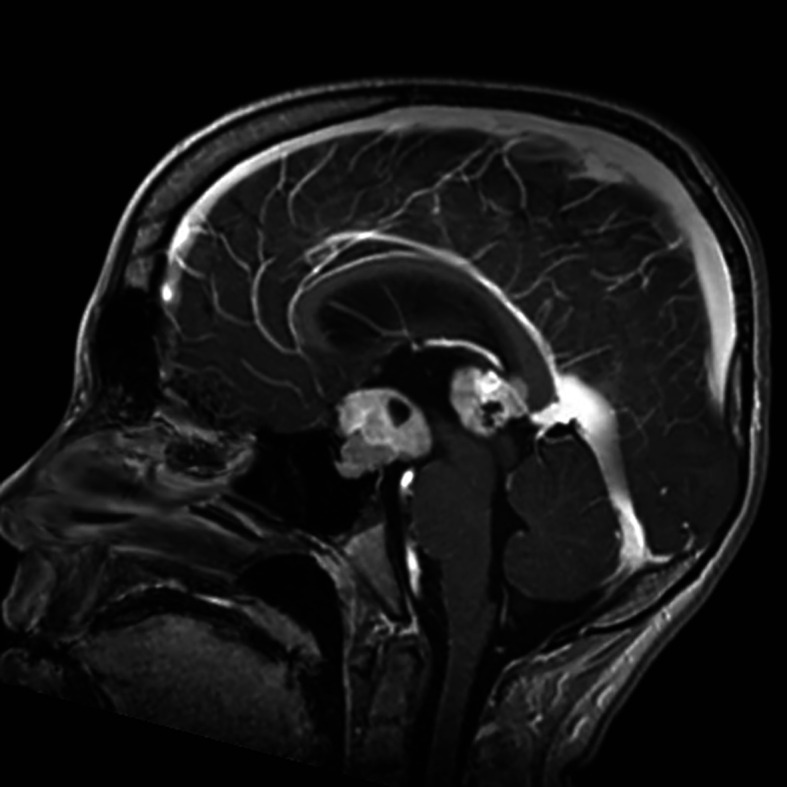
Post-contrast sagittal T1-weighted MRI showing bifocal germinoma.

**Figure 2:**
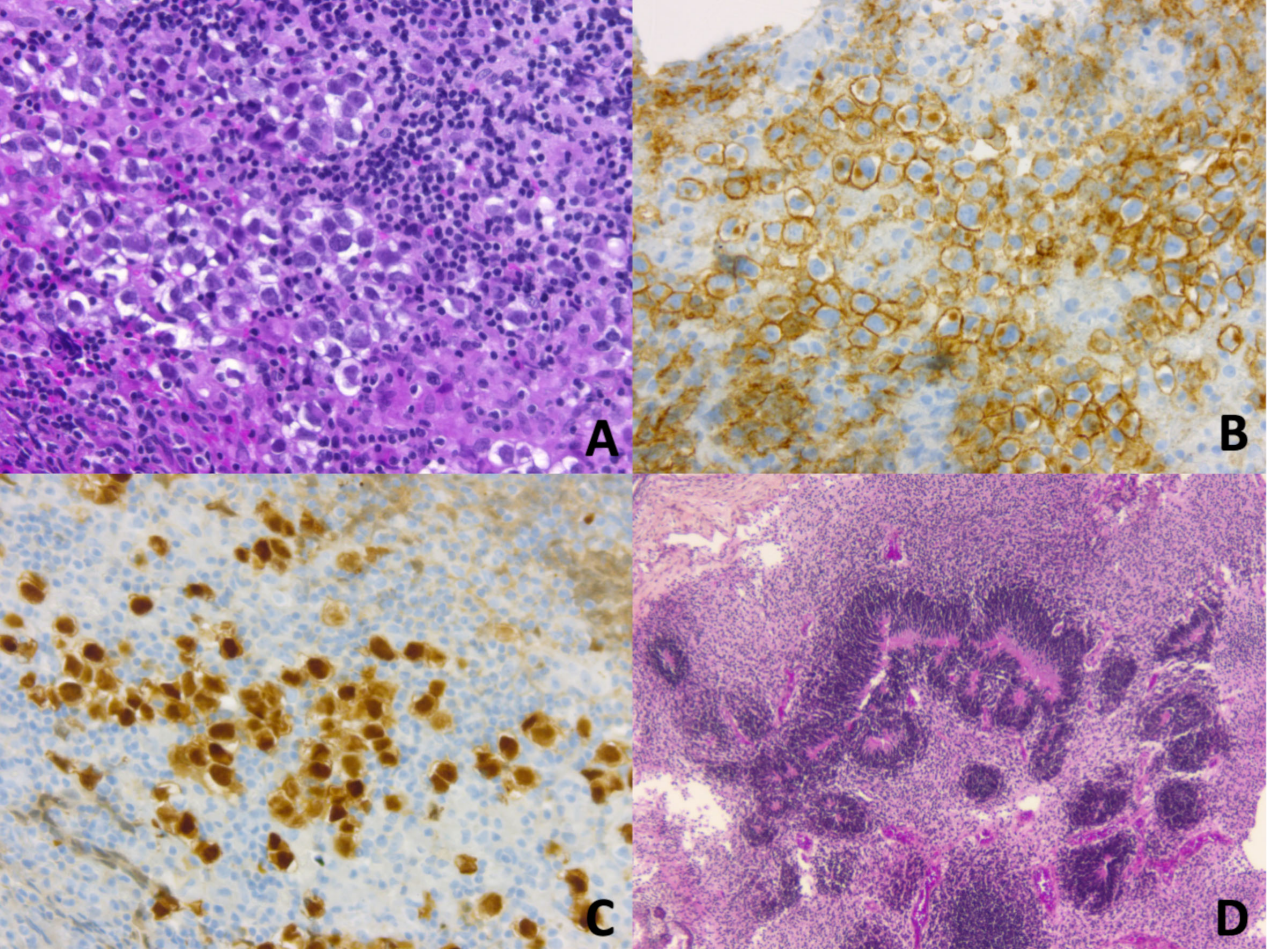
**(A–C)** CNS Germinoma is composed of large round neoplastic cells, with clear cytoplasm, and high nuclear-to-cytoplasmic ratio. They are embedded in an inflammatory stroma of lymphocytes (mostly T cells) and histiocytes (**A**, Haematoxylin, Phloxine, Saffron (HPS) staining, original magnification (OM) x 200). Neoplastic cells demonstrate membranous and golgian expression of CD117/c-Kit **(B**, OM x 200). Nuclei are immunopositive for OCT4 (Octamer-binding transcription factor 4) (**C**, OM x 200). **(D)** CNS immature teratoma is characterized by a variable admixture of fetal and/or embryonic tissues derived from the three germ layers: ectoderm, mesoderm, and endoderm. Rosette-like structures resembling primitive neural tube are typical of immature neuroectodermal components (**D**, HPS staining, OM x 50).

**Figure 3:**
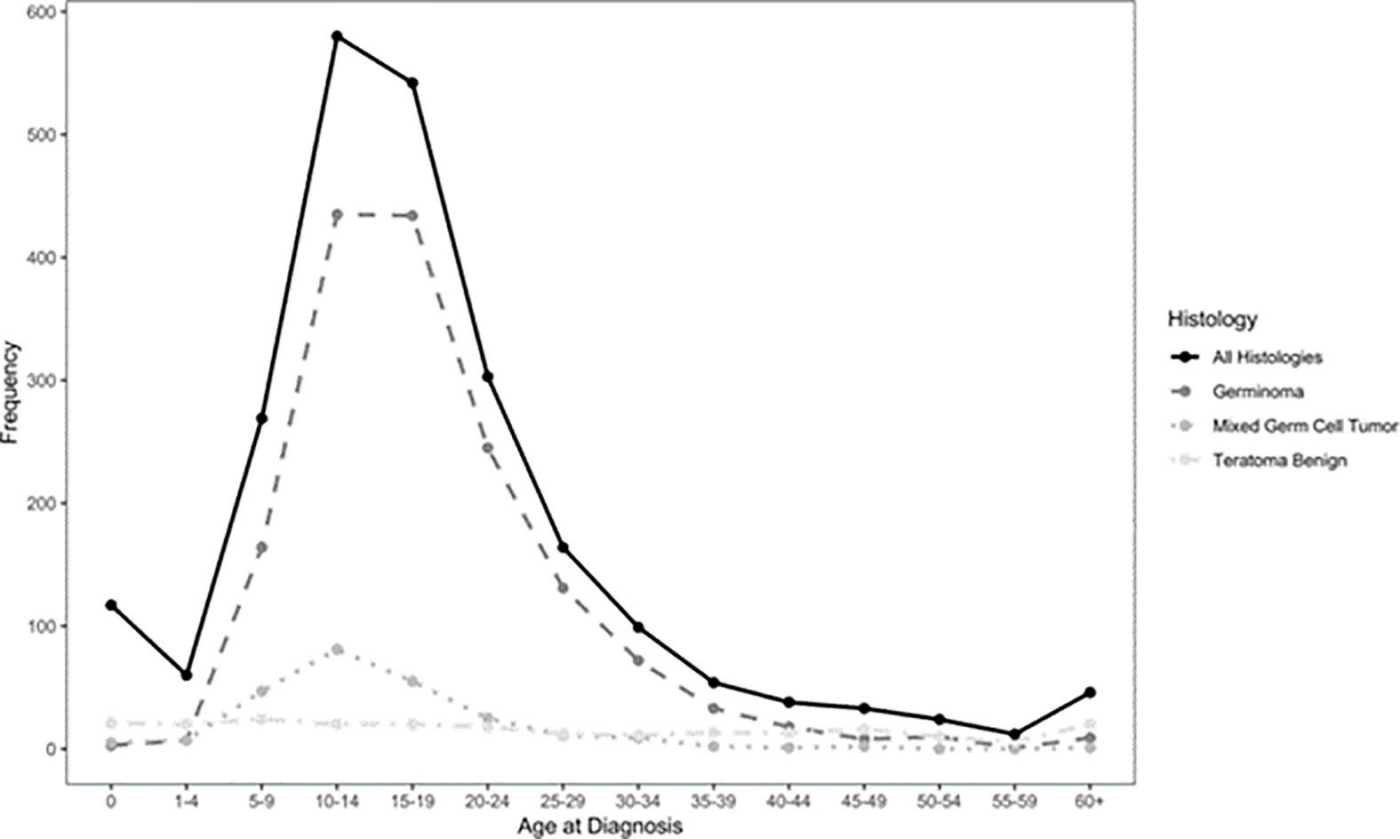
Epidemiology of CNS-GCT (4).

Germ Cell Tumors derive from primordial germ cells, which migrate along the embryo’s midline at five weeks to reach the gonads. Aberrant migration is thought to explain the occurrence of GCTs at extragonadal midline sites. CNS-GCTs develop most commonly in the pineal gland (50%), followed by the suprasellar area (30%). Bifocal tumors (**Figure 1**), defined by the involvement of both the pineal gland and suprasellar area account for 10% of CNS-GCTs and are not regarded as metastatic. Other locations (e.g. the basal ganglia) are uncommon in Western populations, but more frequent in Asia (2). According to the 2021 World Health Organization (WHO) nomenclature (3), GCTs are broadly divided first into germinomas (**Figure 2**), and nongerminomatous GCTs (NGGCTs). Germinoma is twice as common as NGGCT. NGGCTs are subdivided into yolk sac tumor, choriocarcinoma, embryonal carcinoma, mature and immature teratomas, and the collective term “mixed GCT”, used ot describe the combination of at least two GCT subtypes. Among teratomas, “teratoma with somatic-type malignancy” is a very rare entity defined by the malignant transformation of a teratomatous component into a non-GCT malignancy (such as rhabdomyosarcoma). The incidence of CNS-GCT peaks during adolescence and young adulthood, thus representing a model of AYA tumor (**Figure 3**) (4). While CNS-GCTs make up only 0.9% of all CNS primary tumors in the United States, this proportion reaches 3.8% and 3.9% in the 0-14 year and 15-19 year population, respectively (5). The incidence also varies according to the gender and the ethnic background: males are twice more affected as females, and the incidence is 22% higher in the Asian pacific islander population as compared to the white population (5). High incidence persists in East Asian migrants, suggesting a genetic background (6). Development of symptoms can be insidious (for example isolated diabetes insipidus), leading to delayed diagnosis, with one-third of patients with CNS-GCT having more than six months of symptoms prior to diagnosis (7).

## Evolution of treatment approaches for CNS-GCT in Europe

Globally, three main groups, primarily led by pediatric oncologists and/or neurosurgeons, are developing therapeutic strategies for CNS-GCTs: the Japanese pediatric brain tumor study group, the Children’s oncology group (COG), and the International Society of Pediatric Oncology (SIOP). As the response to treatment and the prognosis of NGGCTs are clearly poorer than that of germinomas, therapeutic strategies have evolved separately (**Table 1**). This article focuses on the historical evolution of European CNS-GCT protocols.

**Table 1:** Three approaches to the diagnosis and management of IGCTs.

	GCT type	Diagnostic criteria	Risk group	Treatment
Europe SIOP GCT II (closed to accrual) Upper age limit: no	NGGCT	Pathologic confirmation of CC, YST,CE Or AFP > 25 ng/ml or HCG> 50 UI/L	Localised	PEI chemotherapy followed by surgery of any residue and focal radiotherapy (54 Gy)
			Metastatic	PEI chemotherapy followed by surgery of any residue and CSI (30 Gy) and boost (24 Gy) to primary and metastatic sites
	Germ.	Pathological confirmation of pure germinoma or non secreting bifocal GCT	Localised	CarboPEI followed by WVI radiotherapy (24 Gy) +/- boost to the primary (16 Gy)
			Metastatic	CSI (24 Gy) and boost to primary (16 Gy) and mets
USA- Canada ACNS 1123 (closed to accrual) Upper age limit: 21 years	NGGCT	Pathologic confirmation of CC, YST or CE Or AFP > 10 ng/ml or HCG> 100 UI/L	Localised	CEI chemotherapy followed by WVI (30.6 Gy) and boost to primary (23.4 Gy)
	Germ.	Pathological confirmation of pure germinoma	Localised	CE chemotherapy followed by WVI (18 Gy or 24 Gy depending on response) and boost to the primary (12 Gy)
Japan Japanese Intracranial Germ Cell Tumor Study Group Ongoing trial jRCTs031180223 Upper age limit: no	Germ.	Pathological confirmation of pure germinoma +/- syncytiotrophoblastic giant cell	Good prognosis group	CE chemotherapy followed by WVI (23.4 Gy) WB RT if basal ganglia
	NGGCT	Pathologic confirmation of CC, YST or CE	Intermediate risk • Mixed GCT mainly composed of germinoma or teratomas • Immature teratomas • Teratoma with malignant transformation	CE chemotherapy and concurrent radiotherapy • multiple tumors around 3^rd^ ventricle-WVI (50.4 Gy) • single lesion around 3^rd^ ventricle –focal RT (27 Gy) and WVI (23.4 Gy) • basal ganglia tumor or multiple tumors in the parenchyma – focal RT (23.4 Gy) and WB RT(27 Gy) Post irradiation chemotherapy depending on response
			Poor prognosis • Choriocarcinoma (CC) • Yolk sac tumor (YST) • Embryonal carcinoma (EC) • Mixed tumor mainly composed of CC, YST, or EC • Tumors with AFP ≧ 2000 ng/ml, or HCG ≧ 2000 IU/L	ICE chemotherapy and concurrent focal radiotherapy (30.6 Gy) followed by CSI (30.6 Gy) and ICE chemotherapy

CE, carboplatine and etoposide; CarboPEI, alternating CE and Etoposide Ifosfamide.; CEI, carboplatin etoposide ifosfamide, ICE, ifosfamide cisplatine etoposide; PEI, cisplatine etoposide ifosfamide; CSI, craniospinal irradiation; WVI, whole ventricle irradiation; WB, whole brain; RT, radiotherapy.

**Figure 4:**
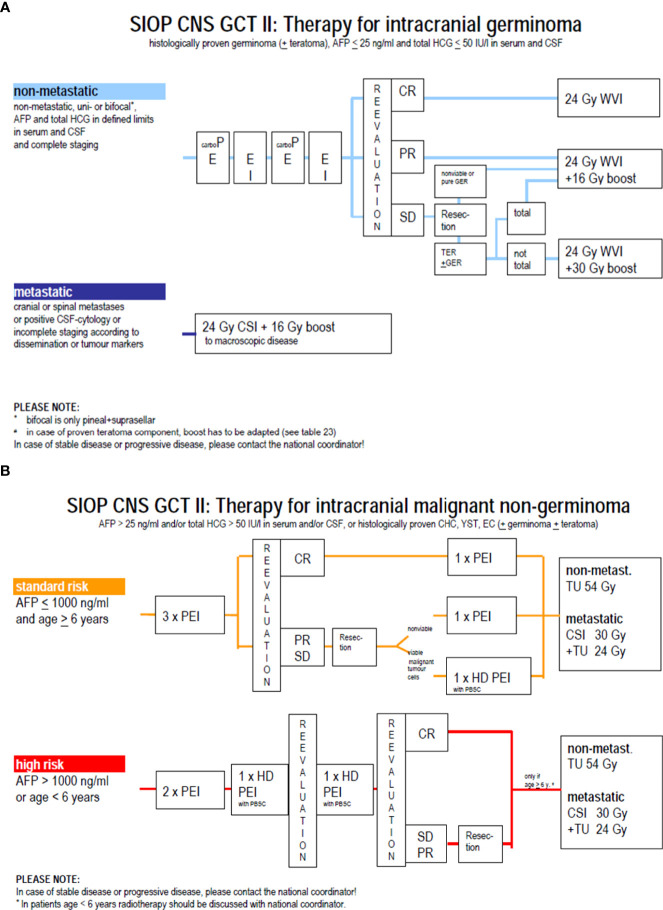
**(A)** Therapy for germinoma in SIOP CNS GCT II protocol. **(B)** Strategy for non germinoma in SIOP CNS GCT II protocol.

For germinomas, craniospinal irradiation (CSI) has long been the gold standard for all stages worldwide, with excellent outcomes but concerns regarding long-term sequelae, such as cognitive deterioration, endocrine dysfunction, and secondary RT-induced tumors (8). The introduction of chemotherapy in the late 80s (9), driven by the high chemosensitivity observed in extracranial GCTs, led to attempts to decrease radiotherapy volume and dose or even to avoid irradiation. However, attempts to avoid radiotherapy altogether resulted in high relapse rates (50%) and unacceptable treatment-related mortality (4/45 patients), making radiotherapy an essential component of multimodality treatment (10). The European experience of 2 national pediatric groups was first reported in 1994; the TC 88 and TC 90 studies conducted by the French Society of Pediatric Oncology (SFOP) and the MAKEI 86 and MAKEI 89 studies led by the German Society of Pediatric Oncology and Hematology (GPOH) (11). The SFOP protocols consisted of combined treatment with chemotherapy followed by radiotherapy, while in the MAKEI protocols, no chemotherapy was given but only radiotherapy. The TC 88 protocol consisted of 2 cycles of chemotherapy with a combination of cisplatin or carboplatin with vinblastine and bleomycin, followed by 30 Gy focal irradiation. Patients with the leptomeningeal disease were treated with CSI to a dose of 20 Gy. The TC 90 protocol consisted of 2 cycles of CarboPEI (carboplatin 600 mg/m² on day 1, etoposide 150 mg/m² on days 1–3, ifosfamide 1.8 g/m² on days 22–26, and etoposide 150 mg² on days 22–24) followed by radiotherapy to the initial tumor volume (40 Gy), and CSI (25Gy) for metastatic disease (12). In the MAKEI 86 protocol, 36 Gy CSI followed by a 14 Gy boost to the tumor site was applied. In MAKEI 89, because of its efficacy, the CSI dose was reduced to 30 Gy (13). Combined treatment (as per SFOP strategy) and exclusive irradiation (as per GPOH strategy) were subsequently incorporated as therapeutic options for non-metastatic germinoma in the non-randomized first European joint protocol SIOP CGT96. The combined treatment (option B) was similar to the TC90 protocol, whereas, in the radiotherapy alone treatment (option A), the CSI dose was reduced to 24 Gy with a 16 Gy focal boost. Metastatic germinoma was treated with either 2 cycles of CarboPEI followed by 24 Gy CSI with a 16 Gy boost or with radiotherapy alone. The key finding of this trial for non-metastatic germinoma (n= 190) was an inferior PFS with option B compared with option A. Indeed, the 5-year PFS was 88 ± 4% and 97% ± 2% in the combined treatment group and the radiation alone group, respectively (p=0.004). Most relapses in option B occurred in the ventricles (14), thus suggesting a role for whole ventricular irradiation. The presence of a post-chemotherapy residue had no impact on prognosis. For metastatic germinoma (n=45), the outcomes with or without additional chemotherapy were excellent, (5-year PFS 100 ± 2%, 5-year OS 100%) thus not justifying the need for chemotherapy in this setting (15). These results paved the way for the subsequent SIOP CNS-GCTII trial, (EUDRACT 2009-018072-33) (**Figure 4A**). In non-metastatic germinoma (including bifocal tumors), the goal was to avoid CSI by using option B (carbo PEI and focal radiotherapy) implemented with 24 Gy whole ventricular irradiation. In case of complete remission after chemotherapy, the 16 Gy boost to the tumor bed was omitted. For metastatic germinoma, treatment consisted of 24 Gy CSI followed by 16 Gy boost to the tumor site and all sites of macroscopic metastatic disease. The trial was closed to accrual in July 2018, and results are pending.

NGGCTs are less sensitive to radiotherapy than pure germinomas, and chemotherapy was rapidly implemented in the therapeutic arsenal with an increase in the cure rate (16). Drugs proved efficient against NGGCT were carboplatin, cisplatin, etoposide, ifosfamide, and bleomycin. In the MAKEI 89 trial, patients received two courses of BEP (bleomycin at 15 mg/m² days 1–3, etoposide at 150 mg/m² days 1–2, and cisplatin 20 mg/m² days 4-8), followed by delayed tumor resection and two courses of VIP (vinblastine 3 mg/m² day 1-2, ifosfamide 1.5 g/m² days 1–5, and cisplatin 20 mg/m² days 1–5). Patients also received CSI at a dose of 30 Gy followed by a focal tumor bed boost of 20 Gy. The 5-year event-free survival was 57% ± 9 (17). During the same period, the SFOP group explored the possibility of avoiding irradiation when a complete response was reached following dose-dense carboplatin-based chemotherapy, but the results were disappointing: 18 patients were enrolled in the TC90 protocol, but 12 of the 13 non-irradiated patients experienced a relapse (18). In the following first joint European SIOP GCT96 protocol, patients received four courses of PEI (cisplatin 20 mg/m^2^ on day 1-5, etoposide 100 mg/m^2^ on day 1-3, ifosfamide 1.5 g/m^2^ on day 1-5), and resection of any post-chemotherapy residue. Patients with the localised disease received focal radiotherapy at a dose of 54 Gy, while those with the metastatic disease received CSI at a dose of 30 Gy CSI followed by a focal dose of 24 Gy. In patients with localised localized NGGCT (n = 116), the study reported a 5-year PFS and a 5-year OS of 72% ± 4 and 82% ± 4, respectively. Initial AFP level (serum and/or cerebrospinal fluid level >1000 ng/mL) and residual disease (defined as any persistent enhancing visible lesions at the tumor site) were independent adverse prognostic factors (19). These results paved the way for the subsequent trial, the SIOP CNS-GCTII protocol (**Figure 4B**), which offers the same strategy (i.e., 4 courses of PEI and a focal or CSI irradiation depending on the stage). However, high-risk patients received high-dose chemotherapy with HyperPEI (cisplatin 20 mg/m^2^ on day 1-5, etoposide 300 mg/m^2^ on day 1-5, ifosfamide 2 g/m^2^ on day 1-5, and autologous stem cell rescue (ASCT) at day 7). High-dose chemotherapy was given in cases of an initial AFP > 1000 ng/ml, age below 6 years (because of the desire to avoid radiotherapy at this age), or persistent viable malignant component at the time of post-chemotherapy resection. The trial was closed to accrual in July 2018, and the results for NGGCT are currently pending.

## Treatment approach for CNS GCTs in AYA

In 1993, a European working group focusing on CNS-GCTs was established by SIOP. The SIOP GCT 96 protocol was further opened for children and adolescents below 18 years of age in 8 countries in Europe and the study accrual spanned January 1996 to December 2005. Three hundred eighty-four patients (235 germinoma and 149 NGGCT) were enrolled, including 30 (8%) adults, despite the upper age boundary set up by the protocol, thus underlining the need for dedicated therapeutic strategy in this population. In France, 56 patients were included, and only 2 were adults (3%).

The SIOP CNS-GCTII was opened in 8 European countries with no upper age limit. Recruitment started in October 2011 and closed to enrolment in 2018. No protocols were open on the “adult side” during the same period.

**Figure 5:**
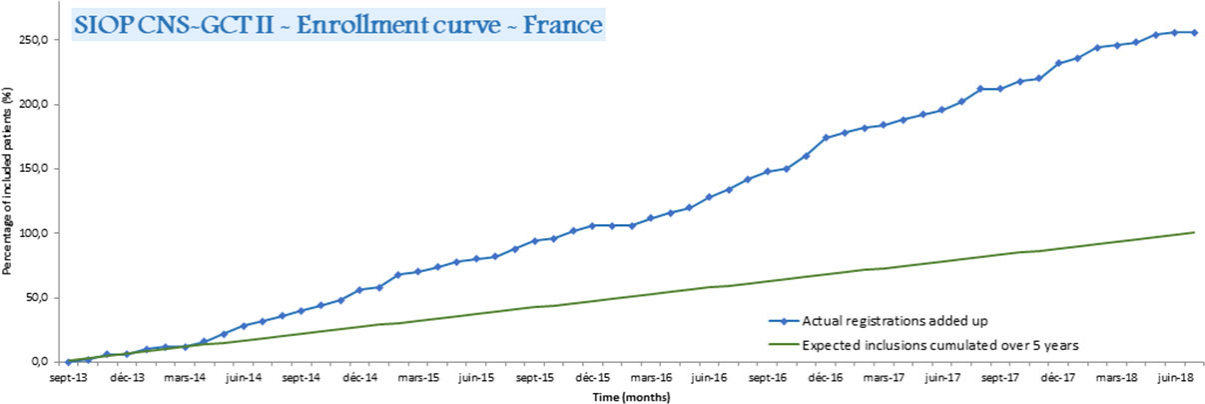
Accrual in the SIOP GCTII protocol in France.

In 2008, a national weekly virtual AYA-dedicated multidisciplinary tumor board (MTB) was launched in France led by the Centre Leon Bérard. Initially, it was dedicated to medulloblastoma, but progressively increased its scope to include any AYA with a brain tumor (20). The number of discussions in this AYA-MTB for CNS-GCTs cases progressively increased from 4 in 2013 to 21 in 2019 (personal data). The SIOP CNS-GCT II protocol was innovative in allowing the inclusion of both adult and pediatric GCT patients. As CNS-GCTs are very rare in adults and as SIOP (among other collaborative groups) had a long history of international collaboration on rare pediatric diseases, it naturally became an attractive model for clinicians treating adults with this orphan disease. Thus, in France, 30% of the patients included in the SIOP GCTII protocol were adults, and 70% of them were included by neuro-oncologists treating adults, which led to higher than expected recruitment (**Figure 5**). This suggests that setting an upper age limit exclusively for an administrative purpose is disadvantageous for patients and, therefore, should be discouraged. In addition, dissemination of information on important clinical research to adult oncologists through meetings and other means of communication could lead to increased rates of inclusion of eligible patients in clinical protocols. This was successfully achieved for CNS-GCTs with the support of the ANOCEF (Association of French-speaking neuro-oncologists).

## Other perspectives in neuro-oncology

Since SIOP CNS-GCTII, other protocols have opened the door between pediatric and adult strategies. The concept of age cut-off has been challenged, as including patients only up to the age of 18 or 21 is an arbitrary decision based more on legislative or logistic grounds than on scientific rationale. A cut-off based on physical development was proposed in medulloblastoma studies. The SIOP PNET5-MB (EudraCT Number 2011-004868-30) includes patients younger than 22 year old at the time of the diagnosis of medulloblastoma. The EORTC 1634-BTG/NOA-23 (EUDRACT 2020-003063-26) consists of both adult and post-pubertal patients with Sonic Hedgehog (SHH) mutation, who may benefit from an experimental treatment by Sonidegib (21). These two protocols thus include a similar sub-cohort of AYA patients, but are not in competition. Rather, they offer the best choice for a subpopulation of patients with SHH-activated medulloblastoma. The limitation is that not all institutions can open both pediatric and adult protocols for each disease.

Another way to extend pediatric concepts to adult neuro-oncology is to correlate unexpected similarities in CNS tumors at various sites through molecular biology. In the future, adults with H3K27M altered gliomas may benefit from the translation of treatment approaches from pediatric diffuse infiltrating pontine glioma (DIPG) of childhood. Pediatric DIPG is a relatively frequent disease in pediatric terms. It has represented a persisting challenge for decades as it is uniformly fatal, despite radiation therapy and multiple trials of chemotherapy. International cooperative efforts are ongoing to modify the current strategy of primary palliative radiation therapy. Biopsies that were previously thought too high risk are currently undertaken more frequently (22). They allow for confirmation of pathology, and provide samples for molecular biology that have contributed to a better understanding of the physiopathology (23). Since the WHO 2016 classification, H3K27M altered gliomas are now recognized as a specific entity (24). A molecular link with infiltrating glial neoplasms of the midline was established both in children and adults. Innovative therapies proposed for DIPG according to their molecular targets could thus be extended to this broader group of neoplasms of the midline. Based on shared biological targets, extension of inclusion criteria to the adult population is now on its way in BIOMEDE protocol (EudraCT Number: 2014-001929-32). Specific MTBs devoted to the treatment of adults with brainstem gliomas are currently running (25). In close contact with pediatricians, the strategy of biopsy followed by targeted therapy and radiotherapy will also now be promoted in adults.

Similarly, proof of concept phase I-II therapies targeted on the identification of a driving mutation may be shared between CNS and non-CNS tumors, and between adult and pediatric patient populations. These targets are involved in mechanisms of carcinogenesis and/or tumor growth but are neither specific to an organ nor to a histologically defined tumor subgroup. Pooling tolerance and efficacy data obtained in pediatric, AYA, and adult populations (26) allowed, for instance, anti-NTRK compounds to obtain rapid EMA accreditation. Here again, collaboration from different scientific societies, both CNS/non-CNS and Pediatric/Adult, provided a sufficient number of patients for an orphan disease to reach the required numbers for validation of this drug. This is a win-win strategy both for pediatric groups to obtain permission to use the drugs in children more rapidly and for adults where these tumors may be rarer.

## Uniqueness of the AYA population and ways to improve their care

Improving care of the AYA population also involves the need to understand how the treatment should be adapted for this particular population. Even where tumor entities in children and adult populations may be the same, children, AYA and adults differ in terms of dosage management, including tolerance to treatment, which may require dose modifications or schedules. For example, more grade IV hematotoxicity and grade II neurotoxicity were reported with the same chemotherapy regimen in patients with medulloblastoma aged 10 to 20 years when compared to children aged 5 to 10 years, thus leading to more frequent dose reductions and more treatment delays in the former group (27). The treatment-related death rate of brain tumor patients receiving high-dose chemotherapy with stem cell rescue was significantly higher in patients older than 18 years than in younger patients (28). The management of psychological and sociological problems is also specific to the AYA population. Schooling is particularly important in this period of life when patients look for their future employment and occupation. Furthermore, their long-term management, considering puberty, requires specific expertise. Thus, though indications for treatment and general management are similar, patients are probably optimally treated either in a pediatric or an adult unit depending on the underlying type of cancer (29).

There are several ways to improve the management of AYAs with cancer and their recruitment into clinical trials. Firstly, there is an urgent need for supportive national policies. In 2014, the third French national cancer plan devoted one of its actions to improving the care of the AYA population. Secondly, the upper age limit to be included in clinical trials should be removed or at least based on a cut off scientifically designed, such as puberty. From a regulatory standpoint, there is no obstacle to include adults in pediatric clinical trials, but the reverse (i.e., including children or adolescents in adult trials) has long been more challenging with regard to ethical concerns (30). European Pediatric Medicine Regulation (1901/2006/EC and 1902/2006/EC) has nevertheless facilitated the access of children to otherwise adult clinical trials and promoted research development in pediatric oncology. However, the existing joint protocol is not sufficient to make meaningful differences, and close collaboration between pediatricians and oncologists is essential. Such collaborations could be set up either by encouraging dialogue between both teams through dedicated MTB, as illustrated with the national AYA brain tumor MTB, but also by scheduling a pediatric session in each adult symposium so that physicians treating adults remain aware of new strategies in some diseases that represent orphan entities in their daily practice. A concerted effort in training stakeholder teams in AYA care is also crucial. For this purpose, a specific national training programme dedicated to AYA care (D.I.U. cancer chez l’adolescent et le jeune adulte- interuniversity diploma for adolescent and young adult with cancer) led by the university of Angers will be launched in 2022 (http://www.univ-angers.fr/fcsante). Moreover, some institutions have already developed specific units for AYA patients, with the attendance of both adult and pediatric specialists who can share their expertise daily (31). At least, there is a need to increase awareness among general practitioners regarding symptoms that should prompt CNS imaging in order to decrease the delay to initial diagnosis (32).

## The ERN-EURACAN project

Launched by the European Commission on 17th March 2017, along with 23 other European Reference Network (ERN) for other rare, complex diseases, EURACAN is dedicated to rare solid tumors and coordinated by the French Comprehensive Cancer Centre Léon Bérard in Lyon, France. EURACAN aims to help spread knowledge on rare cancers through Europe. EURACAN domain 10 especially focuses on rare CNS tumors, and a consensus paper on first-line therapy guidelines for CNS-GCTs has been released recently within its framework (33). For relapsing or refractory CNS-GCTs, there is scant current evidence to help clinicians determine the best therapeutic strategy, and EURACAN thus offers an interface to discuss such complex cases through remote MTB.

## Conclusion

By omitting an upper age limit, the SIOP CNS-GCTII protocol became an attractive model for clinicians treating young adults with this orphan disease. Through dedicated national MTBs, close collaboration between pediatricians and neuro-oncologists has emerged, thus leading to a higher proportion of adults included in clinical trials. It is intended that the EURACAN project, which involves pediatric and adult neuro-oncologists, will be extended to set up European MTBs around difficult cases, hopefully strengthening the benefit already seen in France at the European level.

## Data availability statement

The original contributions presented in the study are included in the article/supplementary material. Further inquiries can be directed to the corresponding author/s.

## Author contributions

CC and DF contributed to conception and design of the study. CC, JN, GC and DF provides data, CC wrote the first draft of the manuscript. JN edited the manuscript. TA, GM, AV wrote sections of the manuscript. All authors contributed to manuscript revision, read, and approved the submitted version.

## Conflict of interest

AI reports research grants from Carthera, Transgene, Sanofi, Air Liquide, Servier, Nutritheragene, advisory board for Leo Pharma, Novocure and Bochringer Ingelhein Int, travel funding from Novocure, Carthera and Leo Pharma outside the submitted work.

The remaining authors declare that the research was conducted in the absence of any commercial or financial relationships that could be construed as a potential conflict of interest.

## Publisher’s note

All claims expressed in this article are solely those of the authors and do not necessarily represent those of their affiliated organizations, or those of the publisher, the editors and the reviewers. Any product that may be evaluated in this article, or claim that may be made by its manufacturer, is not guaranteed or endorsed by the publisher.
